# Implementation of a genotyped African population cohort, with virtual follow-up: A feasibility study in the Western Cape Province, South Africa

**DOI:** 10.12688/wellcomeopenres.23009.1

**Published:** 2024-10-22

**Authors:** Tsaone Tamuhla, Anna K Coussens, Maleeka Abrahams, Melissa J Blumenthal, Francisco Lakay, Robert J Wilkinson, Catherine Riou, Peter Raubenheimer, Joel A Dave, Nicki Tiffin

**Affiliations:** 1 South African Medical Research Council Bioinformatics Unit, South African National Bioinformatics Institute, University of the Western Cape, Cape Town, South Africa; 2 Wellcome CIDRI-Africa, Faculty of Health Sciences, University of Cape Town, Rondebosch, Western Cape, South Africa; 3 Infectious Diseases and Immune Defence Division, The Walter and Eliza Hall Institute of Medical Research, Parkville Victoria, Australia; 4 Department of Medical Biology, The University of Melbourne, Melbourne, Victoria, Australia; 5 Division of Endocrinology, Department of Medicine, University of Cape Town, Rondebosch, Cape Town, South Africa; 6 International Centre for Genetic Engineering and Biotechnology, Cape Town, South Africa; 7 Institute of Infectious Disease and Molecular Medicine, University of Cape Town, Rondebosch, Western Cape, South Africa; 8 The Francis Crick Institute, London, England, UK; 9 Department of Infectious Diseases, Imperial College London, London, England, UK; 10 Department of Pathology, University of Cape Town, Rondebosch, Western Cape, South Africa

**Keywords:** Electronic routine health data, virtual cohorts, African genetic data, H3Africa Illumina micro-array, genotype data, tiered informed consent, population admixture, resource-limited environments

## Abstract

**Background:**

There is limited knowledge regarding African genetic drivers of disease due to prohibitive costs of large-scale genomic research in Africa.

**Methods:**

We piloted a cost-effective, scalable virtual genotyped cohort in South Africa, with participant recruitment using a tiered informed consent model and DNA collection by buccal swab. Genotype data was generated using the H3Africa Illumina micro-array, and phenotype data was derived from routine health data of participants. We demonstrated feasibility of nested case control genome wide association studies using these data for phenotypes type 2 diabetes mellitus (T2DM) and severe COVID-19.

**Results:**

2267346 variants were analysed in 459 participant samples. 78.6% of SNPs and 74% of samples passed quality control (QC). Principal component analysis showed extensive ancestry admixture in study participants. For 1780 published COVID-19-associated variants, 3 SNPs in the pre-imputation data and 23 SNPS in the imputed data were significantly associated with severe COVID-19 cases compared to controls. For 2755 published T2DM associated variants, 69 SNPs in the pre-imputation data and 419 SNPs in the imputed data were significantly associated with T2DM cases when compared to controls.

**Conclusions:**

The results shown here are illustrative of what will be possible as the cohort expands in the future. Here we demonstrate the feasibility of this approach, recognising that the findings presented here are preliminary and require further validation once we have a sufficient sample size to improve statistical significance of findings.

We implemented a genotyped population cohort with virtual follow up data in a resource-constrained African environment, demonstrating feasibility for scale up and novel health discoveries through nested case-control studies.

## Plain language summary

What are we researching?

Our study focuses on understanding the genetic causes of diseases in African populations, where there is very little genetic data available. Even though it's cheaper now to gather genetic data, it's still expensive to collect large amounts of data needed to study these populations properly.

What were the aims of the research?

Create an African Genotyped Cohort: This means gathering genetic information from African participants using tools made specifically for African genetics.

Use Strong Informed Consent: We wanted to make sure participants understood and agreed to the study, allowing us to grow the cohort over time.

Use Existing Health Data: To make the process affordable, we used health data that was already being collected.

What did we do?

We made sure our consent process was clear and thorough.

We collected DNA samples from consenting participants using cheek swabs and existing blood samples.

We generated and checked the quality of the genetic data.

We tested if we could study diseases like Type 2 Diabetes and severe COVID-19 with this approach.

What did we find?

In our pilot study, we genotyped 459 samples, with 74% being good quality. We analysed over 2 million genetic markers, with 78.6% passing quality checks.

Our study shows that this approach works well in African populations and can analyse their genetic diversity. The next step is to expand the cohort for more comprehensive studies.

## Introduction

Despite recent efforts to increase the amount of African genomic data ^1– 4^ there is still a marked underrepresentation of African populations in genomic research ^5, 6^. At the same time Africa is undergoing an epidemiologic shift and is experiencing an exponential increase in the prevalence of non-communicable diseases (NCDs) like Type 2 diabetes mellitus (T2DM) ^7, 8^. While it is well known that NCDs like T2DM are caused by a combination of lifestyle and genetic factors ^9– 12^, most African studies have focused on the lifestyle drivers of these diseases ^13– 16^ and there remains limited knowledge on their genetic drivers in African ancestry population.

While the costs of generating human genomic data have reduced significantly in recent times, in Africa, they are still a barrier to the large-scale implementation of genomic research ^17– 21^. Single nucleotide polymorphism (SNP) genotyping is a widely used, cost-effective method of generating large scale genomic data. Previously available micro-array genotyping chips, however, were not always optimal for identifying disease associated variants in African ancestry populations ^6, 22^. The recent availability of the Infinium™ H3African Consortium V2 array (H3Africa chip) ^23^ which contains novel African variants has now made it possible to generate more informative genotype data for genome wide association studies (GWAS) in African genomes.

Since GWAS identify the association of genotypes with phenotypes, it is critical to ensure that phenotype definition is accurate and generated in a standardised way to avoid introducing bias and spurious associations ^24, 25^. While electronic medical records have been used elsewhere as a readily available cost-effective resource for generating GWAS phenotypes ^26– 33^ they are currently not widely available in African countries. The increasing availability of electronically captured and curated patient routine health data in African health systems ^34– 36^, however, presents an opportunity to use these data in African genomic studies. In addition, we have previously demonstrated how routine health data can be modelled to describe patient phenotypes at both an individual and population level ^37, 38^.

Given NCDs are predicted to surpass infections as the leading cause of morbidity and mortality in African populations by 2030 ^7, 8^ and that the genetic risk of diseases in African populations cannot be accurately predicted with the existing resources, there is an urgent need to perform large scale genomic research in Africa.

In order to be fully informative, agile and responsive, disease-agnostic longitudinal cohorts representing the general population can provide a wealth of research data ^39^, for both existing and emerging health challenges. They can also be very costly to set up and maintain depending on the existing infrastructure ^40, 41^. The work presented here is intended as a proof-of-concept study demonstrating that a genotyped virtual cohort linking routine health data and genotype data is feasible and economical for generating new research outputs and understanding disease aetiology in less-resourced environments, particularly in Africa. The cohort is described as virtual because participant interaction will only happen once when they are enrolled into the study with informed consent. The longitudinal prospective health data we collect do not require any further interaction with participants as this will be done virtually from electronic routine health data. While the concept of virtual cohorts is new, the International epidemiology Databases to Evaluate AIDS (IeDEA) consortium have shown that it is feasible in the African context ^42^.

 In this pilot study, we demonstrate the following principles: firstly, that genotype data can be generated from buccal swabs from consenting participants; secondly, that the genotype data can be successfully linked to longitudinal routine health data from the department of health, to create a prospective cohort without the need for follow-up visits; thirdly that these data are appropriately structured for conducting nested case control studies that can validate known aetiological variants or identify novel variants; and overall, that we have successfully created a prospective, disease-agnostic genotyped cohort that is affordable to grow and maintain in a resource-constrained environment.

## Methods

### Study population and sampling

All adults (18 years or older at recruitment) who consented to access of their clinical data from the Provincial Health Data Centre (PHDC), a health information exchange containing routine health data for about 7 million healthcare clients, collated daily from multiple electronic health data sources in the Western Cape Province, South Africa ^34^ and to give a DNA sample were eligible for participation in the study. For some participants, DNA samples were collected using buccal swabs and the buccal swab method was chosen because it is relatively inexpensive, easy to administer, non-invasive and the samples are easy to store and transport after collection ^43^. The buccal swab participants were recruited from health care clients visiting diabetes day clinics at Groote Schuur Hospital, Cape Town, South Africa and two swabs were collected from each consenting participant.

***DNA Extraction from Buccal Swabs.*** DNA samples for genotyping were prepared from the buccal swabs at an external commercial facility (Central Analytic Facility, Stellenbosch University) to allow for the optimisation of DNA preparation protocols that can be scaled for large sample sizes. Genomic DNA was extracted from buccal swabs using the Macherey-Nagel NucleoSpin® Tissue kit, with modifications. Swabs were placed in 15 mL falcon tubes, followed by the addition of 800 µL 1x PBS and 50 µL Proteinase K. After vortexing, tubes were incubated at 56°C for 10 minutes. The liquid was transferred to 2 mL Eppendorf tubes and any remaining liquid was extracted by inverting the tubes.

Following the manufacturer’s protocol, 600 µL of Buffer B3 and 600 µL ethanol were added, and the lysate was loaded onto NucleoSpin® Tissue columns in 600 µL increments. The columns were washed with Buffer BW and Buffer B5, with final centrifugation steps to remove ethanol. DNA was eluted in 40 µL of Buffer BE and samples were stored at -20°C until further analysis.

***DNA Quantification and Quality Assessment.*** DNA concentration and purity were initially assessed using a NanoDrop™ spectrophotometer, aiming for 260/230 ratios of 2.0–2.2 and a 260/280 ratio of ~1.8. Samples with concentrations below 60 ng/µL were concentrated by evaporation at 56°C for 30 minutes.

For precise quantification, the Qubit™ dsDNA High Sensitivity Assay Kit was used. Qubit reagent and buffer were mixed in a 1:200 ratio to prepare the working solution. Standards and samples were prepared by adding 190 µL of working solution to each tube, followed by 10 µL of standards or 1 µL of DNA sample. Tubes were vortexed briefly and incubated at room temperature in the dark for 2 minutes. DNA concentration was measured using the Qubit 4 Fluorometer, and results were recorded.

***Collaborative Model.*** In addition, since we were piloting a study design that would allow the cohort to grow over time including through collaborations with other studies, we piloted a collaborative recruitment strategy where different groups with consenting participants can collaborate and combine their DNA samples for genotyping in the same batch to increase sample size and reduce the cost. To test this approach, DNA extracted from peripheral blood samples of consenting participants (age >18 years) from the HIATUS (HIV and Tuberculosis in SARS-CoV2) study was included for genotyping in this study. Ethical approval for this study was granted by the Faculty of Health Sciences Human Research Ethics Committee (HREC) at the University of Cape Town (Approval Number: HREC 207/2020). Initial approval was obtained on 22 ^nd^ May 2020, with annual renewals thereafter. The informed consent process was administered by a recruitment field officer. Participants who consented to take part in the study were asked to sign a written consent form, which was also signed by recruitment staff as witnesses to the process. Written consent was therefore obtained from all participants, and a copy of the signed form was provided to each participant for their records. Participants recruited into the HIATUS study included two arms, 1) hospitalized COVID-19 diagnosed and non-COVID-19 controls presenting at Groote Schuur Hospital, Cape Town, South Africa investigating the clinical and immunological interaction between COVID-19, HIV, TB and other co-morbidities ^44, 45^; and 2) asymptomatic TB household contacts and community controls who were longitudinally followed up for TB ^46^ who consented for extended follow-up for TB or COVID-19 diagnosis during the COVID-19 pandemic in 2020-21.

***DNA Extraction from EDTA Peripheral Blood.*** Genomic DNA was extracted from EDTA-treated peripheral blood using the QIAamp DNA Blood Midi Kit (QIAGEN), following the manufacturer’s protocol without modifications. Briefly, the recommended volume of blood was processed, and lysis was achieved using Buffer AL and Proteinase K, followed by incubation. The lysate was applied to the QIAamp Midi spin column, and DNA was purified through a series of wash steps with Buffers AW1 and AW2. Finally, DNA was eluted in Buffer AE and stored at -20°C until further use. DNA concentration and purity were estimated using the NanoDrop spectrophotometer.

### Genotyping

DNA samples from 459 unrelated study participants were genotyped on the Infinium™ H3Africa Consortium Array V2 (H3Africa chip), a custom genotyping chip with ~2.26 million SNPs including novel African variants ^23^. Array BeadChips were analysed on the Illumina iScan™ System and the GenomeStudio™2.0 Genotyping Module was used to make genotype calls and generate PLINK PED and MAP files ^47^ from the raw genotype data.

### Genotyping quality control

Quality control (QC) of the genotyped data was done in PLINK1.9 ^47^ using the protocol from Marees and colleagues ^48^ with some modifications. QC was done for both samples (n = 459) and SNPs (n = 2 267 346).

*Sample QC*

Samples with a genotype call rate of less than 98% were excluded from the dataset. Discordant sex information is when the recorded sex and the genotype sex do not match and for this analysis sex imputation using the genotype sex was done for samples with discordant sex information ^48^. All samples failing sex imputation were excluded from the dataset. Samples were checked for relatedness and those with an identity by descent (IBD) score of more than 0.2 were also excluded ^48^. Finally, samples with an outlying heterozygosity score (more than 3 standard deviations from the mean) were also excluded from the dataset ^48^.

*SNP QC*

SNPs with a missingness of more than 2%, a Hardy-Weinberg equilibrium p-value less than 1 x10 ^-6^ or a minor allele frequency (MAF) less than 1% were all excluded from the dataset. A MAF threshold of 1% was used instead of the widely used 5% because the H3Africa chip contains some variants that occur with a MAF =< 1% in African populations ^23^ and the same threshold has been applied in a recent study ^49^ which also used genotype data from the H3Africa chip. In addition, all non-autosomal SNPs (X, Y and mitochondrial) were also excluded from the data during QC ^48^.

### Population stratification

The structure of the participant population was assessed using multi-dimensional scaling (MDS) and principal component (PC) analysis in PLINK1.9 ^47^ following the protocol from Marees and colleagues ^48^. Prior to conducting the MDS and PC analysis, the data were first LD pruned leaving only a subset of uncorrelated SNPs which were then merged with the 1000 Genomes data which contains well defined reference populations. Following the analysis, 10 MDS components were extracted as covariates to be used in the genome wide association analysis to control for population stratification bias. MDS and PCA plots were generated using R statistical software ^50^.

### Imputation

Prior to phasing and imputation, reference allele mis-match was checked and any problematic data subsequently corrected and normalised using BCFtools ^51^, an open access tool. Phasing using EAGLE2 ^52^ and genome wide imputation using positional Wheeler-Burrows transform (PWBT) ^53^ were then done on the Sanger Imputation Service using the African Genome Resources reference panel ^54^ both of which are open access. Post imputation QC included the removal of indels, poorly imputed SNPs (Info score <= 0.6) and SNPs with a MAF <= 1 resulting in a final dataset with 342 participants and ~13.55 million SNPs.

## Nested case-control GWAS

### Identification of cases and controls

To demonstrate the feasibility of carrying out a GWAS with these data, two nested case-control GWAS (T2DM and severe COVID-19) were done using both the pre-imputation and post-imputation quality-controlled data. This was undertaken as a proof-of-principle analysis recognising that the studies would not have sufficient statistical power to generate decisive results. The cases and controls for the two studies were identified using phenotype data inferred from the PHDC routine health records of the participants. A T2DM case was inferred from PHDC records using listed disease evidences of at least one glycated haemoglobin (HbA1c) value greater than or equal to 6.5% ^55^ and/or dispensed diabetes drugs as previously described ^38^. A severe COVID-19 case was also inferred from PHDC records using listed disease evidences of a positive SARS-CoV-2 polymerase chain reaction (PCR) laboratory result and hospital admission for the treatment of SARS-CoV-2 infection as previously described ^37^. For either phenotype case-control study, the participants who did not meet the defined case criteria were then treated as controls.

### GWAS

Since the phenotypes in both studies were binary, a logistic regression GWAS using the 10 MDS components as covariates was done in PLINK1.9 ^47^. Quantile-quantile (QQ) plots were plotted in R to check for biases in the data which if not controlled for could result in erroneous false positive associations ^48^. Manhattan plots were also plotted in R to identify SNPs with the strongest associations based on -log10 of their p-values.

### Identification of known T2DM and COVID-19 variants

To do a descriptive analysis in participants for known disease-associated variants previously identified in other populations, T2DM (3524) and COVID-19 (2916) associated SNPs were downloaded from NHGRI-EBI GWAS Catalog ^56^ on 17/03/2024 and compiled into lists. Using PLINK1.9 ^47^ SNPs from the lists were identified in the study dataset and extracted from the genotyped data, and their allelic counts and associated odds ratios calculated to determine their occurrence in the cases compared to the controls for each disease phenotype.

### Statistical analysis

Summary statistics were calculated for the study population using R version 3.6.3 ^50^. For continuous data, median and interquartile range were calculated and for grouped data, percentages were calculated. For median values, the Wilcoxon rank sum test was used to calculate significance of differences between groups; and significance of the differences in proportions between groups was tested using the Fisher’s exact test. The Bonferroni correction was applied to adjust for multiple testing.

## Results

### DNA quality from buccal swabs

We collected two buccal swab samples from 61 consenting participants and the DNA was extracted and stored separately for each sample. DNA from 49 (80.3%) participants was successfully extracted from both buccal swabs while in 12 (19.7%) participants only 1 of the buccal swabs gave an adequate DNA sample. Qubit quantification of the extracted DNA gave a median concentration of 36.9 (IQR: 23.0, 57.5) ng/μl and total median DNA yield of 2.79 (IQR: 1.88, 4.27) μg per sample. In addition, the highest DNA yields were obtained from participant self-administration of the swab under clinician supervision (data not shown). By comparison, DNA from blood gave a median concentration of 38.9 (IQR: 29.9, 57.4) ng/μl and total median DNA yield 1.95 (IQR: 1.50, 2.57) μg per sample.

### Genotyping quality control

The total genotype call rate for the study was 97.0% before QC. A total of 1782023 (78.6%) SNPs and 343 (74%) samples with a final total genotype call rate of 99.9% passed QC and were available for further analysis. When comparing the QC pass rate by sample type, a higher proportion of buccal swab samples (80.3%) (49/61) passed QC compared to 73.8% (294/398) of blood samples ( Table 1) and most of the excluded samples (n= 95) were due to excessive genotype missingness at a 2% threshold. For individual SNPs, 12.6% (n= 285975) of the genotyped SNPs were excluded because they had a MAF less than the 1% threshold.

**Table 1.** Characteristics of the study participants in the severe COVID-19 nested case-control GWAS.

	All (n = 343)	Controls (n = 280)	Severe COVID-19 cases (n = 63)	p-value
Sex:				<0.001
Male	114 (33.2%)	80 (28.6%)	34 (54.0%)	
Female	229 (66.8%)	200 (71.4%)	29 (46.0%)	
Age (years)	45.0 [35.0;56.0]	43.0 [34.0;54.0]	54.0 [44.0;64.5]	<0.001
DNA sample:				0.842
Blood	294 (85.7%)	241 (86.1%)	53 (84.1%)	
Buccal swab	49 (14.3%)	39 (13.9%)	10 (15.9%)	

### Population structure

**Figure 1.**
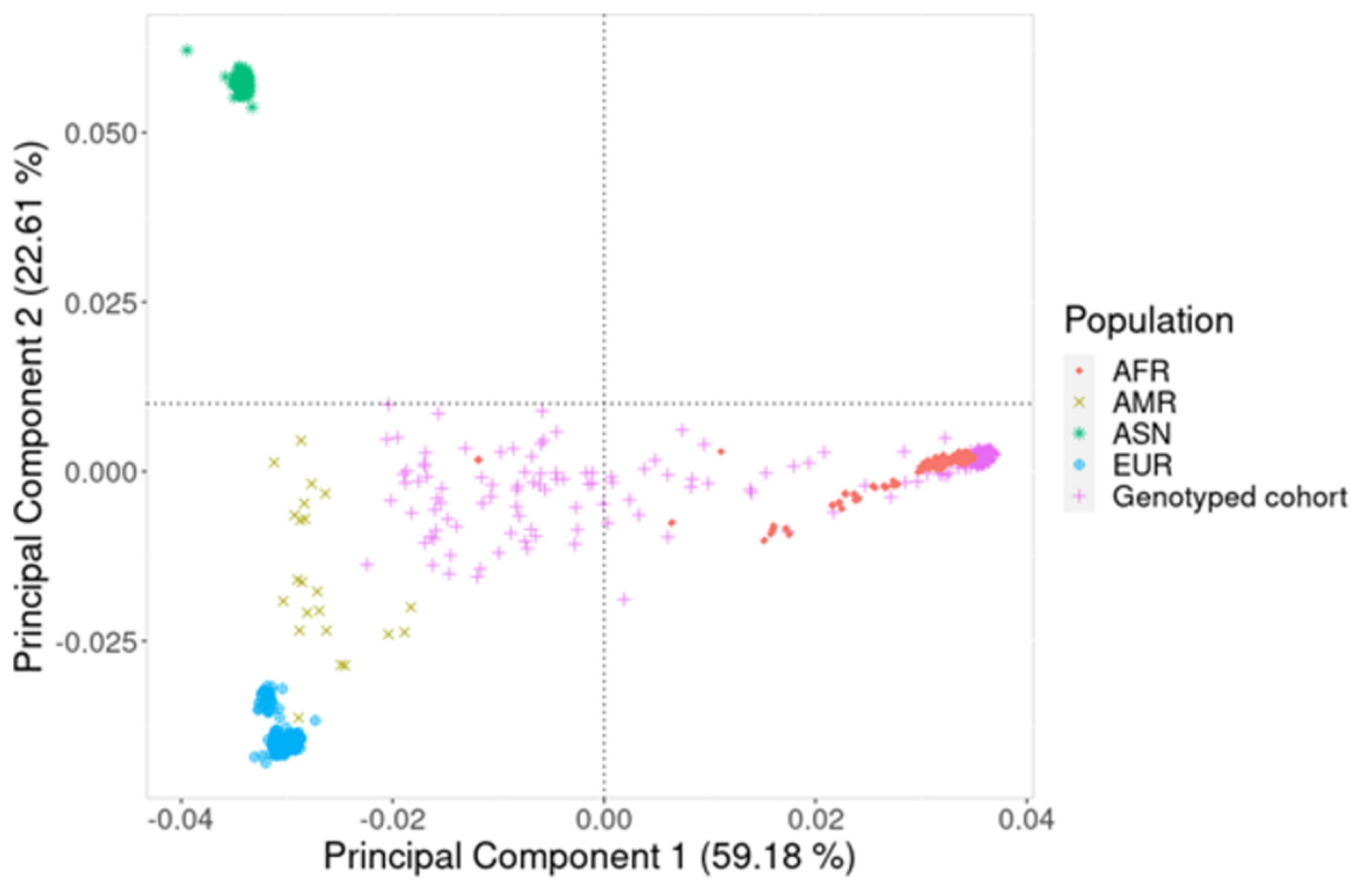
Principal component (PC) analysis plot using PC1 and PC2. These two components show the genetic variation of the study participants in comparison to well defined continental populations in the 1000 Genomes dataset. AFR (♦) is African, AMR (x) is American, ASN ( _*_) is Asian, EUR (⊕) is European and Genotyped cohort (+) is the study participants from the Western Cape Province, South Africa.

An analysis of the population structure using PCA ( Figure 1) and MDS (Additional file 1) showed extensive admixture in the study participants. This is because while some of the study participants clustered with the African population in the 1000 Genomes data, a significant proportion did not form clusters and were spread across the plot, showing extensive genetic variation.

### Study participants in the nested case-control GWAS

The 343 participants whose samples passed QC were majority female 66.8% (n = 229) and had a median age of 45 years [IQR: 35, 56] ( Table 1). When looking at each case-study separately, there were 63 cases of severe COVID-19 identified and 280 controls ( Table 1). When comparing cases and controls there were significant differences (p< 0.001) in sex distribution and the controls had a high proportion of females (71.4%) whereas the cases were mostly male (54.0%) ( Table 1). Similarly, there were significant differences (p<0.001) in median age between cases and controls. The cases had a median age of 54.0 years [IQR: 44.0, 64.5] compared to a younger control population of 43.0 years [IQR: 34.0, 54.0] ( Table 1). There was no significant difference (p=0.842) between the cases and controls based on DNA extracted from buccal swab or blood in the severe COVID-19 GWAS ( Table 1).

For the T2DM GWAS, 93 cases and 250 controls were identified from the 343 samples that passed QC ( Table 2). While the controls had a higher proportion of females (69.2%) compared to the cases (60.2%), there were no significant differences (p=0.149) in the sex distribution between the cases and controls ( Table 2). There were however significant differences in age (p-value < 0.001) with the controls being younger with a median age of 40.5 years [IQR: 32.0, 50.0] while the median age for the cases was 57.0 years [IQR: 49.0, 65.0]. There were also significant differences in the DNA samples that generated the genotype data for the cases and controls. All the controls were from blood samples whereas the cases were genotyped from almost equal proportions of blood and buccal swab samples ( Table 2). Since the genotyping was done in the same batch, there is no need to control for the DNA sample type.

**Table 2.** Characteristics of the participants in the T2DM nested case-control GWAS.

	All (n = 343)	Controls (n = 250)	T2DM Cases (n = 93)	p-value
Sex:				0.149
Male	114 (33.2%)	77 (30.8%)	37 (39.8%)	
Female	229 (66.8%)	173 (69.2%)	56 (60.2%)	
Age (years)	45.0 [35.0;56.0]	40.5 [32.0;50.0]	57.0 [49.0;65.0]	<0.001
DNA sample:				<0.001
Blood	294 (85.7%)	250 (100.0%)	44 (47.3%)	
Buccal swab	49 (14.3%)	0 (0.0%)	49 (52.7%)	

### Severe COVID-19 GWAS

**Figure 2.**
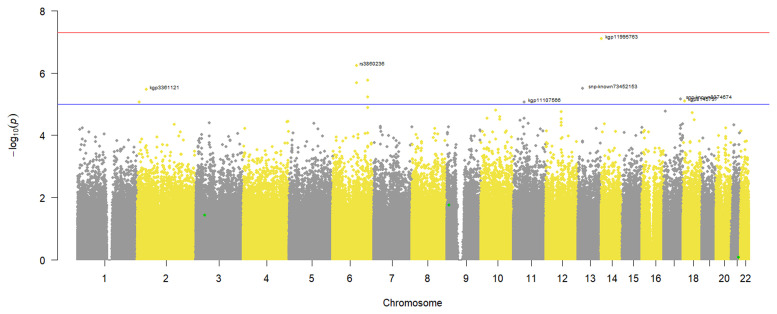
Manhattan plot of a severe COVID-19 case-control GWAS in 343 individuals from a virtual genotyped cohort in the Western Cape Province, South Africa. The genome wide significant threshold (5x10-8) is shown by the top horizontal line and the suggestive threshold (1x10-5) by the bottom horizontal line. The following annotated SNPs; kgp3361121, rs38660236, kgp11107566, kgp11995763, snp-known73452153, snp-known8074674 and kgp8145737 crossed the suggestive threshold. The SNPs highlighted in green represent published COVID-19 variants that were identified in the pre-imputation quality control dataset.

Since the phenotype was binary, a logistic regression GWAS was done to identify variants associated with severe COVID-19 in our study participants. From the results in Figure 2, no genotyped variants in the pre-imputation dataset were associated with severe COVID-19. This is because none had a p-value that crossed the genome wide significance threshold of 5x10 ^-8^. However, 11 SNPs crossed the suggestive threshold p-value of 1x10 ^-5^ ( Figure 2). In addition, the annotated SNPs on the Manhattan plot had p-values smaller than the suggestive threshold, and when multiple SNPs on the same chromosome cross the threshold only the one with the smallest p-value is annotated ( Figure 2). Similarly, no variants in the imputed dataset were associated with severe COVID-19 in the study participants (Additional file 2).

**Figure 3.**
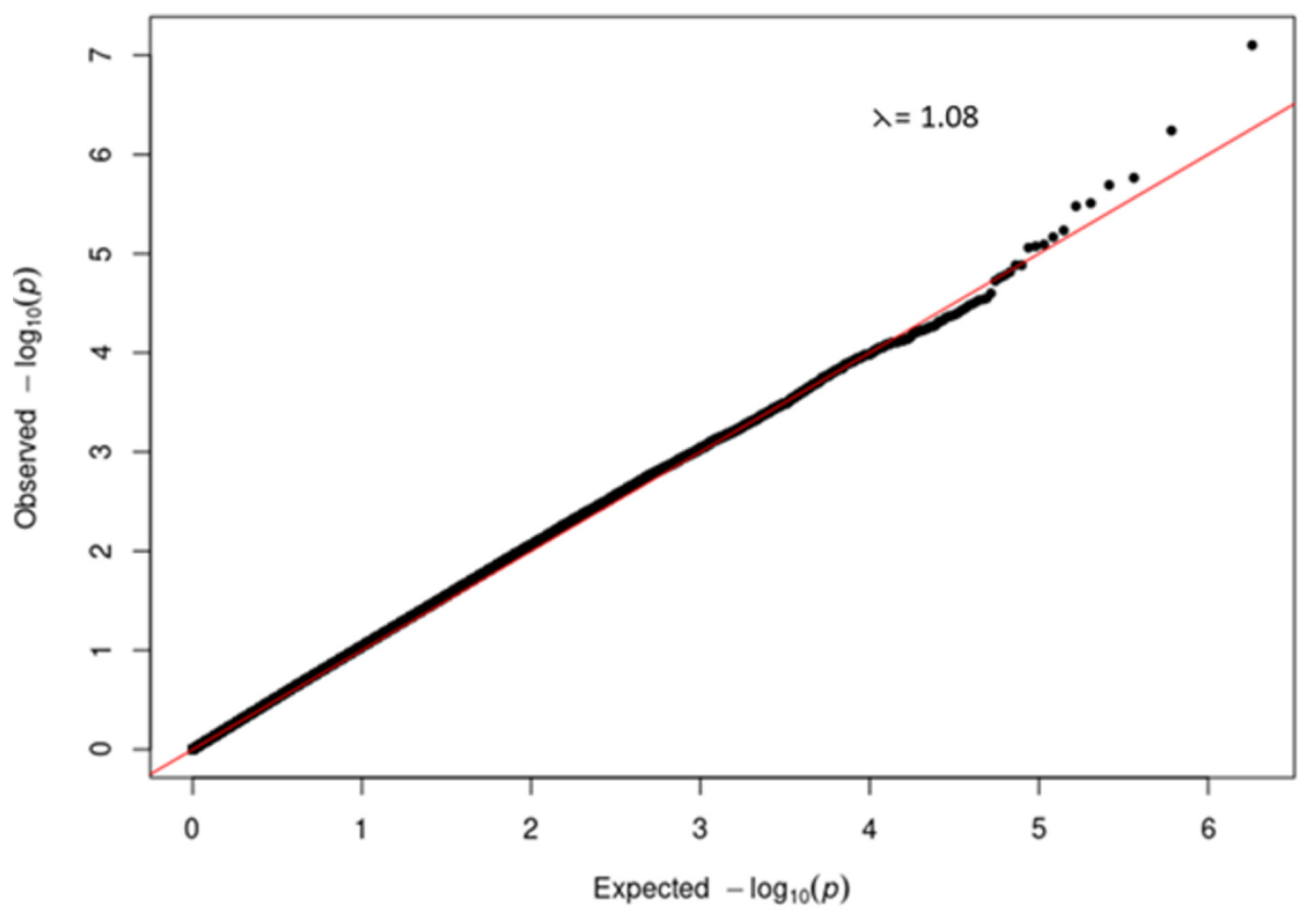
Quantile-quantile (QQ) plot of -log10 p-values from the severe COVID-19 GWAS Manhattan plot.

A quantile-quantile (QQ) plot ( Figure 3) of the -log _10_ p-values from the Manhattan plot in Figure 2 showed that the observed associations were not confounded by the population admixture ( Figure 1) that is present in the study participants.

### T2DM GWAS

**Figure 4.**
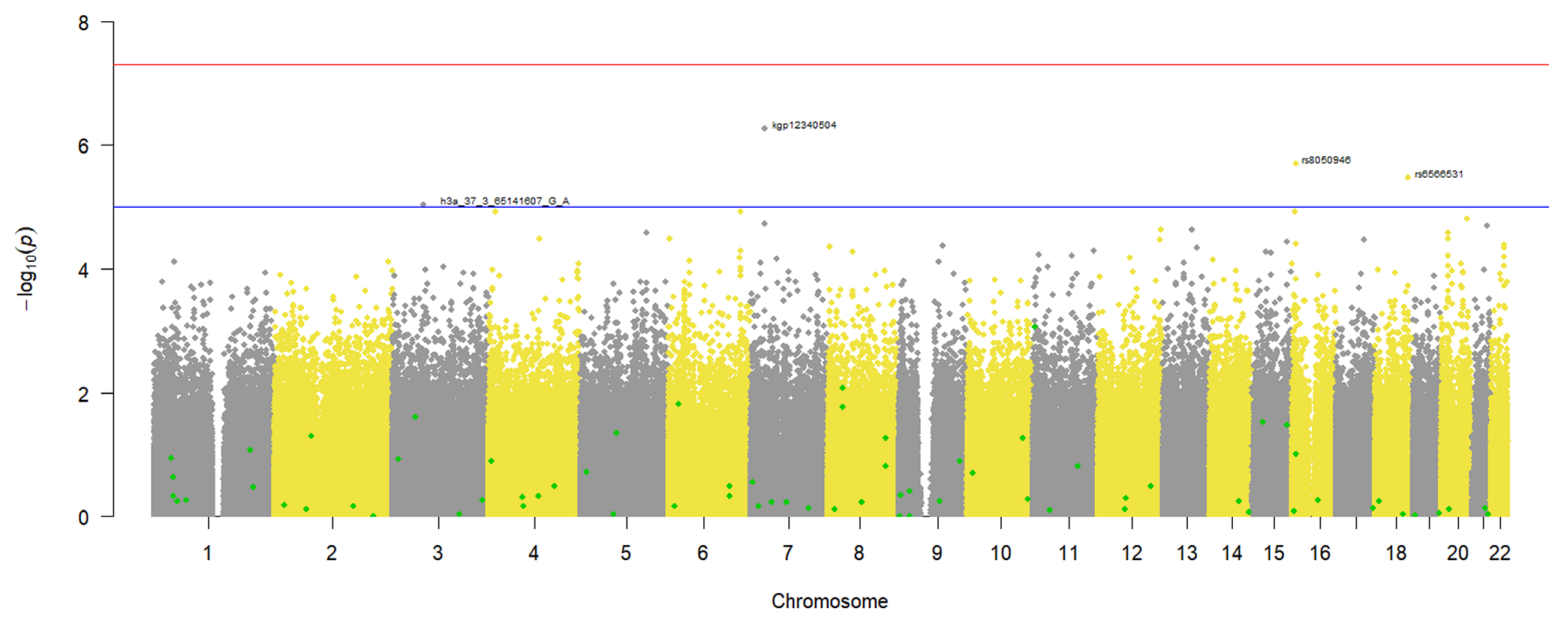
Manhattan plot of the T2DM case-control GWAS in 343 individuals from a virtual genotyped cohort in the Western Cape Province, South Africa. The suggestive threshold (1x10-5) is shown by the horizontal line and the following annotated SNPs; h3a_37_65141607_GA, kgp12340504, rs8050946 and rs6566531crossed it. The SNPs highlighted in green represent known T2DM variants that were identified in the pre-imputation quality control dataset.

Since the phenotype was binary, a logistic regression GWAS was done to identify variants associated with T2DM in our study participants. From the results in Figure 4, no genotyped variants in the pre-imputation dataset were associated with T2DM. This is because none had a p-value that crossed the genome wide significance threshold of 5x10 ^-8^. However, 4 SNPs crossed the suggestive threshold p-value of 1x10 ^-5^ and are annotated on the plot ( Figure 4). Similarly, the GWAS using the imputed dataset (Additional file 3) did not identify any variants associated with T2DM in our dataset.

**Figure 5.**
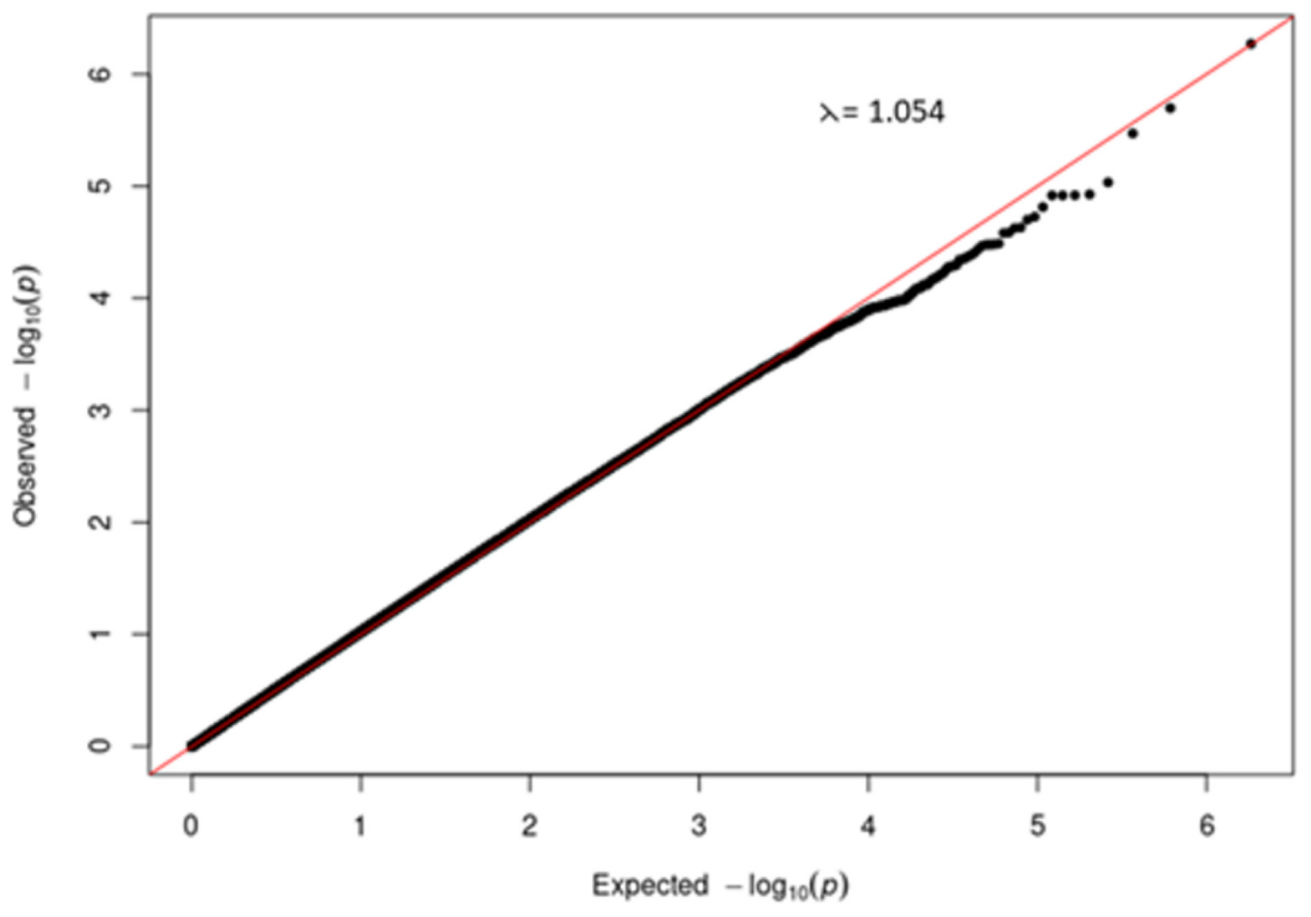
Quantile-quantile (QQ) plot of -log _10_ p-values from the T2DM GWAS Manhattan plot.

A quantile-quantile (QQ) plot ( Figure 5) of the -log _10_ p-values from the Manhattan plot in Figure 4 showed that the observed associations were not confounded by the population admixture ( Figure 1) that is present in the study participants.

### Identification of known COVID-19 associated variants

*Pre-imputation dataset*

A total of 165 published COVID-19 variants were identified in the pre-imputation dataset and a comparison of their occurrence in severe COVID-19 cases and controls identified 50 SNPs associated with reduced odds of occurring in severe COVID-19 cases (odds ratio < 1), however after adjusting for multiple-testing these odds were not statistically significant. The other 115 SNPs were associated with increased odds of occurring in COVID-19 cases and of these only three occurred in significantly higher counts in the severe COVID-19 cases than controls in the study participants: rs4818059 (OR: 3.07, 95% CI: 1.75-5.37, p-value = 0.007), rs10815977 (OR: 2.25, 95% CI: 1.50-3.39, p-value = 0.012) and rs3796187 (OR: 2.78, 95% CI: 1.62-4.77, p-value = 0.022) ( Table 3). These 3 COVID-19 associated variants identified in the study participants are highlighted in green on the GWAS Manhattan plot ( Figure 2) and after the multiple testing adjustment in this genome wide hypothesis testing, they are not highlighted as potential aetiological variants. A larger sample size would be required to make reliable conclusions in this scenario.

**Table 3.** Allele counts of statistically significant published COVID-19 associated variants identified in the genotyped cohort pre-imputation data.

SNP	Nearest gene	Base pair location ^a^	Risk allele ^b^	Risk allele counts ^c^ Cases (n= 63)	Risk allele counts ^c^ Controls (n = 280)	Odds ratio	95% CI ^d^	p-value unadjusted ^e^	p-value adjusted ^f^
**rs4818059**	B3GALT5	Chr 21, 40942788	A	22	40	3.07	1.75–5.37	4.48 x 10 ^−5^	0.007
**rs10815977**	PTPRD	Chr 9, 8860355	A	48	133	2.25	1.50–3.39	7.09 x 10 ^−5^	0.012
**rs3796187**	DCLK3	Chr 3, 36779707	A	23	47	2.78	1.62–4.77	1.34 x 10 ^−4^	0.022

^a^Location of the SNP on the genome denoted by chromosome number and base pair position. ^b^Alleles aligned to genome build GrCh37. ^c^count of 0 if not risk allele, 1 if heterozygous for risk allele and 2 if homozygous for risk allele. ^c^95% confidence interval. ^d^Fisher’s exact test p-value. ^e^Bonferroni adjusted p-value.

*Imputed dataset*

A total of 1780 published COVID-19 associated variants were identified in the imputed dataset and a comparison of their occurrence in cases and controls identified 594 SNPs with reduced odds of occurring in severe COVID-19 cases. After correcting for multiple testing, none occurred in significantly lower counts in severe COVID-19 cases than controls in the study participants. The other 1186 SNPs were associated with increased odds of occurring in COVID-19 cases and of these 23 occurred in significantly higher counts in the severe COVID-19 cases than controls in the study participants (Additional file 4).

### Identification of known T2DM variants

*Pre-imputation dataset*

A total of 292 published T2DM variants were identified in the pre-imputation dataset and a comparison of their occurrence in T2DM cases and controls identified 88 SNPs associated with reduced odds of occurring in T2DM cases (odds ratio < 1) and after correcting for multiple testing, 5 occurred in significantly lower counts in T2DM cases than controls (Additional file 5). The other 204 SNPs were associated with increased odds of occurring in T2DM cases and of these, 64 occurred in significantly higher counts in the T2DM cases than the controls after correcting for multiple testing (Additional file 5).

In addition, the 69 statistically significant T2DM associated variants identified in the study participants were highlighted on the GWAS Manhattan plot ( Figure 4) but after the multiple testing adjustment in this genome wide hypothesis testing, they are not highlighted as potential aetiological variants. A larger sample size would be required to make reliable conclusions in this scenario.

*Imputed dataset*

A total of 2755 published T2DM associated variants were identified in the imputed dataset and a comparison of their occurrence in cases and controls identified 594 SNPs with reduced odds of occurring in T2DM cases (Additional file 7). After correcting for multiple testing, 17 occurred in significantly lower counts in the T2DM cases than controls in the study participants (Additional file 7). The other 2161 SNPs were associated with increased odds of occurring in T2DM cases and after adjusting for multiple testing, 402 SNPs (Additional file 7) occurred in significantly higher counts in T2DM cases than control participants in the study.

Additionally, 27 SNPs from an African ancestry GWAS ^57^ were identified in the imputed dataset, and after adjusting for multiple testing, only rs887400 (OR:6.07, 95% CI:3.77-9.73, p-value = 0.004) ( Table 4) occurred in significantly higher counts in T2DM cases than controls in the study participants. The 419 statistically significant T2DM associated variants including rs887400 identified in the study participants were highlighted in green on the GWAS Manhattan plot ( Figure 5) but after the multiple testing adjustment in this genome wide hypothesis testing, they are not highlighted as potential aetiological variants. A larger sample size would be required to make reliable conclusions in this.

**Table 4.** Allele counts of published T2DM associated variants from African ancestry populations identified in the genotyped cohort imputation data.

SNP	Nearest gene	Base pair location ^a^	Risk ^b^ Allele	Risk allele counts ^c^ (n= 93)	Risk allele counts Controls ^c^ (n = 250)	Odds Ratio	95% CI ^d^	p-value unadjusted ^e^	p-value adjusted ^f^
**rs9847133**	LMCD1-AS1	Chr 3, 8250522	C	71	188	1.018	0.72–1.44	0.9195	0.9715
**rs887400**	PRELID3BP3	Chr 17, 60945840	G	56	33	6.07	3.77–9.73	4.586e–16	0.004055
**rs80268399**	DLG2	Chr 11, 84152134	C	1	10	0.2638	0.03–2.07	0.1737	0.6303
**rs7903146**	TCF7L2	Chr 10, 114758349	T	64	179	0.9349	0.65–1.33	0.7089	0.8949
**rs77741372**	AP3B1	Chr 5, 77532068	C	10	21	1.291	0.59–2.79	0.5166	0.8185
**rs7747641**	LINC00574	Chr 6, 170418905	T	2	4	1.342	0.24–7.39	0.7342	0.9044
**rs76859863**	LINC00385	Chr 13, 30756207	A	16	57	0.7282	0.41–1.30	0.2838	0.7045
**rs7534008**	LINC01707	Chr 1, 69048029	T	51	182	0.6559	0.45–0.95	0.02502	0.4278
**rs74927455**	ST18	Chr 8, 53032943	A	4	33	0.3097	0.11–0.89	0.0213	0.4152
**rs74073568**	FYB2	Chr 1, 57260359	A	25	61	1.112	0.67–1.83	0.6757	0.8823
**rs7364276**	RPL35P8	Chr 22, 49481929	C	26	86	0.7785	0.48–1.25	0.3008	0.7142
**rs73284431**	AGMO	Chr 7, 15434230	C	10	26	1.031	0.49–2.18	0.9354	0.9771
**rs60227687**	GUCY2EP	Chr 11, 76385728	A	10	41	0.6333	0.31–1.29	0.2057	0.6543
**rs57261374**	RNU6–857P	Chr 18, 28423906	C	22	75	0.7566	0.45–1.26	0.2809	0.7028
**rs4253735**	PPARA	Chr 22, 46611026	G	4	11	0.973	0.31–3.10	0.9631	0.9869

^a^Location of the SNP on the genome denoted by chromosome number and base pair position. ^b^Alleles aligned to genome build GrCh37. ^c^count of 0 if not risk allele, 1 if heterozygous for risk allele and 2 if homozygous for risk allele. ^c^95% confidence interval. ^d^Fisher’s exact test p-value. ^e^Bonferroni adjusted p-value.

## Discussion

In this analysis we conducted a feasibility study demonstrating the implementation of a scalable and cost-effective genotyped virtual population cohort suitable for doing genomic research in under resourced settings. While similar cohorts have been set up elsewhere ^43, 58– 61^ the proposed virtual cohort differs in that it does not require complex infrastructure for biobanking large collections of study samples. This is because the tiered informed consent model used provides an option to recontact participants for future studies where more complex samples and data might be needed. Therefore, a participant only needs to give a DNA sample once and does not need to return for follow-up visits, thus working to also reduce participant research fatigue and loss to follow up.

We demonstrated that the virtual cohort design is an inclusive model which can incorporate collaborators from different research environments if appropriate participant informed consent is in place. We successfully piloted genotyping samples from different studies in the same batch and showed that data generated from samples from different tissue-types and extraction protocols can be combined; and the use of routine health data from a single source and captured using appropriate health informatics standards can facilitate immediate meta-analyses without having to go through a harmonisation process ( Table 1 and Table 2). This means that the cohort is, by design, primed to encourage collaborations with other research programmes in the Western Cape Province, which will both benefit those researchers seeking to add a genomic component to their research, and contribute to reaching a critical mass for statistically sound hypothesis-generating research using the full study population.

In this feasibility study the SNP and sample QC thresholds were adopted from a protocol using European ancestry data ^48^ and we acknowledge that the thresholds used may not have been optimal for data generated from the H3Africa chip ^3^. In particular, instead of the proposed 5% MAF threshold48 for SNP QC, we modified the protocol and used a MAF threshold of 1% in line with other studies conducting genotyping QC on African samples ^49, 62^. As more samples get genotyped on the H3Africa chip we expect to be able to optimise suitable QC thresholds that will provide high quality data for African GWAS.

It is well established that African ancestry populations including those in our genotyped cohort ( Figure 1) are genetically diverse ^63– 66^. The study was set in Western Cape Province which has a highly heterogenous population ^63^, and this heterogeneity was observed in the population structure analysis which showed significant stratification and admixture ( Figure 1). We were able to demonstrate ( Figure 3 and Figure 5) that genetic and genomic analyses done with this cohort can, with the appropriate analysis tools, accommodate the enormous genomic variety in the population of the Western Cape, which has ancient and modern non-admixed and highly admixed African populations, as well as admixture from Europe and Asia ^63, 66– 68^.

While we did not have an adequate sample size for statistically significant GWAS, we were able to demonstrate that the virtual genotyped cohort design can be used successfully for both hypothesis-generating research and hypothesis-testing research. In this study, we were able to successfully identify both African-specific novel SNPs ( Figure 2 and Figure 4) as well as previously known COVID-19 and T2DM aetiological variants ( Table 3 and Table 4, Additional file 4, 5, 6 and 7). We have also demonstrated how the whole cohort may be repurposed successfully for analysis of different diseases, by the design of nested case control studies that stratify the total sample by different disease criteria. This clearly demonstrates how the cohort may be used as a disease-agnostic resource that can address many different disease outcomes. This also means the cohort design is research-agile and can be very responsive to new health challenges that arise. This agility was also demonstrated by existing population cohorts during the current COVID-19 pandemic ^69^ but this work was notable in its lack of African representation ^70^. As it grows over time, the cohort we are building will be able to close this gap in the future. In addition, this virtual cohort model will be even more pragmatic and less expensive to develop than traditional cohorts and health and demographic surveillance systems (HDSS), because there is no need to collect new datasets as the existing ones can be rapidly updated from the routine health data which is updated daily at the PHDC.

Through this analysis we have described how we conceptualised and implemented a genotyped virtual population cohort in a resource constrained environment. We have shown that routine health data can be effectively linked with genotyped data in a GWAS and while acknowledging the small sample size in this feasibility study, we have demonstrated that the H3Africa chip is fit for purpose and can highlight African specific variants. In addition, we have shown that this cohort is able to easily incorporate participants from other studies as long as the appropriate consent is in place, thus paving the way for future collaborations with other research programmes. We are confident that this design and implementation are appropriate to scale up the cohort to a size where novel health discoveries can be made through nested case-control studies.

## Ethics and consent

Ethics approval was granted by the Faculty of Health Sciences Human Research Ethics Committee (HREC) at the University of Cape Town (Approval number: HREC 509/2019). Initial approval was obtained on 30th September 2019, with annual review and renewals thereafter. Permission to conduct the study at Groote Schuur Hospital diabetes clinics and access participant routine health data was granted by the Western Cape Government Health (WCGH), South Africa. A tiered informed consent model was used ^71, 72^. The informed consent process was administered by a recruitment field officer. Participants who consented to take part in the study were asked to sign a written consent form, which was also signed by recruitment staff as witnesses to the process. Written consent was therefore obtained from all participants, and a copy of the signed form was provided to each participant for their records. The participant information and consent forms were in provided in both English and isiXhosa to ensure that language was not a barrier for prospective participants. Genomic data was de-identified using bar-coding of consent forms and collection tubes prior to recruitment and sample collection. Using the barcoded consent forms with the recorded Clinicom folder number, the Provincial Health Data Centre (PHDC, WCGH) ^34^ then linked clinical records to the barcode number using the clinical folder number and returned de-identified data with only the barcode as an identifier. This facilitated linkage of data without overt exposure of personal participant details adds an additional layer of privacy protection, even though permission was provided by participants for the use of their identified data. Data transfer was effected through secure platforms using AES256 encryption and password protection, and analysis was undertaken on a secured, firewall-protected server.

## Data Availability

***Genomic data*** This manuscript provides a proof of concept for setting up the genotyped virtual cohort, using pilot data, and as such the datasets are not yet complete, and primary studies using these data have not yet been undertaken. For this reason these data are not yet openly available. Researchers wishing to undertake collaborative research relating to these data can contact Professor Nicki Tiffin by email at: ntiffin@uwc.ac.za ***Routine health data*** The routine health data were provided for analysis by the Western Cape Department of Health, Provincial Health Data Centre. These are highly granular health data linked to individual health care clients in the province. For this reason, the Western Cape Department of Health does not permit open sharing but instead grants only primary use permission for the data. Re-use of this dataset requires approval from the Western Cape Department of Health (Provincial Health Data Centre), and Dr Moodley, Director: HIA, Western Cape Department of Health, South Africa can be contacted to advise on this process (email: melvin.moodley@westerncape.gov.za, Reference study ID 259-TIFFIN). Open Science Framework (OSF): Virtual Cohort for African MultiMorbidity (VCAMM). https://doi.org/10.17605/OSF.IO/NV3B5 ^73^ This project contains the following extended data: °  Additional file 1.tif °  Additional file 2.tif °  Additional file 3.tif °  Additional file 4.csv °  Additional file 5.csv °  Additional file 6.csv °  Additional file 7.csv °  English_VCAMM_participant_information_and_consent.pdf °  Figure_Legends _Additional_Files.docx Data are available under the terms of the Creative Commons Attribution 4.0 International license (CC-BY 4.0).

## Software availability

- BCFtools is open-access software available at: https://samtools.github.io/bcftools/.
- EAGLE2 is open-source and can be accessed at: https://data.broadinstitute.org/alkesgroup/Eagle/.
- The Sanger Imputation Service using the African Genome Resources reference panel can be accessed at: https://imputation.sanger.ac.uk/?panel=agr.
