# Supplementary material for: Implementation of a genotyped African population cohort, with virtual follow-up: A feasibility study in the Western Cape Province, South Africa
Source: Wellcome Open Res. 2025 Jan 13;9:620. Originally published 2024 Oct 22. [Version 2] doi: 10.12688/wellcomeopenres.23009.2 (PMC11806245; doi:10.12688/wellcomeopenres.23009.2)
Supplement: Peer review history

# Reviewer report: Reviewer response for version 2

**DOI:** 10.21956/wellcomeopenres.25981.r117227

**Published:** 2025-02-10

**Authors:** Maik Pietzner

**Affiliations:** 1 Computational Medicine, Berlin InsJtute of Health at Charité – Universitätsmedizin Berlin, Berlin, Germany

The study by Tamuhla et al. describes an important effort to increase genomic diversity in a low-cost setting. The authors genotyped about 450 participants with existing linkage to electronic health records, a strategy very successfully pursued in multiple countries already. The paper itself is largely descriptive and I missed some important information, mostly centred around the way the authors aimed to demonstrate biological plausible insights from such a small scale study (see more detailed comments below). This is somewhat distracting form the overall effort and appears premature.

 General:

1. I am not an expert, but the term ‘African genetic drivers’ is easily misused and language along the lines of ‘Causative genetic variants more frequent in populations from continental Africa are underexplored’ might be more suitable.
2. There is redundant text (e.g., in the methods in the abstract), also how methods are presented.
3. What explains the high rate of samples not passing QC?
4. The replication of previously published SNPs for T2D and COVID-19 is somewhat odd, if the emphasize is on African genomes and the goal to identify ancestry-enriched signals. Why did the authors not use health outcomes with clear prior evidence of ancestral enriched risk alleles like some kidney diseases or sickle cell anaemia? Or maybe even HbA1c if sufficiently recorded in the health care data?
5. I strongly appreciated the effort the authors have went through to set up this pioneering study in a low-cost setting, but generating reliable genetic data from buccal swabs is not really a novel achievement of the study.
6. A more detailed description of the quality of health records and possibly references to comparisons to gold-standard-diagnostics would be beneficial. I appreciate that many recent Western efforts opted for a similar opportunistic approach, but it would still be good to understand early on, to what extend differential disease ascertainment may drive divergent genetic findings.
7. Not sure whether this is also an issue in South Africa, but how do the researchers ensure that possible sex-mismatches between reported and genotyped sexes do not lead to any adverse consequence of participants?
8. Why was MDS and PCA done? Can the authors recapitulate in MDS plots, that 1kG populations are sufficiently separated? Please make sure, that you settle for either and not use both techniques interchangeably. The population structure should also be described in more detail. For example, how does it correlate with self-identified ancestry?
9. The replication analysis done by the authors lacks some rigour. There is no guarantee that the SNPs that replicate (at a too lenient threshold) do really tag the lead signal at locus or are rather a result of random overlap. For example, how many other SNPs in the GWAS of the SA cohort did meet a threshold of p<0.05 in a 1Mb window around regional lead signals of the previous GWAS, but are clearly no related to the disease risk?
10. Please do not report suggestive p-values. Those findings have almost never replicated in larger studies.
11. The distinction between imputed and genotyped data is artificial and both section should be consolidated to simplify the manuscript.
12. The T2D finding of rs887400 is somewhat odd. The variant is frequent and associated with an almost absurd odds ratio. This usually implies that there is something wrong with the underlying data. It would have been also beneficial to report associated statistics from the well-powered discovery statistics and allele frequencies.

Is the work clearly and accurately presented and does it cite the current literature?

Partly

If applicable, is the statistical analysis and its interpretation appropriate?

Partly

Are all the source data underlying the results available to ensure full reproducibility?

Yes

Is the study design appropriate and is the work technically sound?

Partly

Are the conclusions drawn adequately supported by the results?

Partly

Are sufficient details of methods and analysis provided to allow replication by others?

Partly

Reviewer Expertise:

Genetic epidemiology, statistics

I confirm that I have read this submission and believe that I have an appropriate level of expertise to state that I do not consider it to be of an acceptable scientific standard, for reasons outlined above.

# Reviewer report: Reviewer response for version 2

**DOI:** 10.21956/wellcomeopenres.25981.r117225

**Published:** 2025-02-07

**Authors:** Alexander J Mentzer, Amanda Chong

**Affiliations:** 1 University of Oxford, Oxford, England, UK

Tamuhla, Tiffin and others have presented a proof-of-concept study whereby they have undertaken human genotyping from a combination of buccal swabs and blood samples collected as part of ongoing studies in South Africa. Most of the DNA was available from samples in the HIATUS study, whereas others were available from the PHDC. The authors demonstrate the feasibility of generating genetic data and running simple genetic analyses with traits including severe COVID-19.

 Overall this is a well lain out study. The authors clearly acknowledge the limitations of their statistical design given the small number of cases and control available. Their steps of analyses are generally clear and appropriate and in line with what would be expected for larger roll out of the project. I do have a few comments:

 1) It is difficult from the current description to determine the clinical definitions of hospitalised COVID-19 and T2DM. Were these defined from both cohorts? For hospitalised COVID-19, do you know whether these cases received supplemental oxygen or not? This latter question has relevance for the likelihood if identifying associated variants.

 2) The PCA plot does suggest structure, I agree. Do the authors have any information on self-reported ethnicity / ancestry - this may suggest more differential ancestry rather than admixture per se?

 3) There is a high failure rate with DNA extraction from both blood and saliva. This should be discussed in the Discussion as it has impacts on feasibility. I would expect closer to 10% failure. What do the authors think may be driving this and are there any plans to improve this?

 4) Furthermore, the impact of sample type on genotyping quality could be further explored and discussed as it could have an impact as the cohorts are expanded.

 5) What are the plans for expansion of the resource - are facilities available to support the expansion in the future and what level of phenotypes may be available - this could be a very exciting resource for the area.

 Then minor points include:

 a) could the authors specify the lambda value they think is acceptable. I usually aim for a lambda less than 1.05, but there are publications that accept <1.1

 b) The abstract has duplication:

 "We piloted a scalable virtual genotyped cohort in South Africa that was affordable in this resource-limited context, cost-effective, scalable virtual genotyped cohort in South Africa, with participant recruitment using a tiered informed consent model and DNA collection by buccal swab. "

 Remove the duplicated reference to "scalable virtual genotyped cohort in South Africa"

 In the same sentence: add that some samples were genotyped from blood samples or that multiple sources were used for DNA collection

 I acknowledge my colleague Dr Amanda Y Chong in co-reviewing this manuscript.

Is the work clearly and accurately presented and does it cite the current literature?

Yes

If applicable, is the statistical analysis and its interpretation appropriate?

Yes

Are all the source data underlying the results available to ensure full reproducibility?

Yes

Is the study design appropriate and is the work technically sound?

Yes

Are the conclusions drawn adequately supported by the results?

Yes

Are sufficient details of methods and analysis provided to allow replication by others?

Yes

Reviewer Expertise:

Human genetics

We confirm that we have read this submission and believe that we have an appropriate level of expertise to confirm that it is of an acceptable scientific standard.

# Reviewer report: Reviewer response for version 2

**DOI:** 10.21956/wellcomeopenres.25981.r117147

**Published:** 2025-01-27

**Authors:** Emilio Di Maria

**Affiliations:** 1 University of Genoa, Genoa, Italy

The authors consistently responded to the remarks.

Is the work clearly and accurately presented and does it cite the current literature?

Yes

If applicable, is the statistical analysis and its interpretation appropriate?

Yes

Are all the source data underlying the results available to ensure full reproducibility?

Yes

Is the study design appropriate and is the work technically sound?

Yes

Are the conclusions drawn adequately supported by the results?

Partly

Are sufficient details of methods and analysis provided to allow replication by others?

Yes

Reviewer Expertise:

Clinical Genetics, Genetic Epidemiology, Health Technology Assessment, Health Equity.

I confirm that I have read this submission and believe that I have an appropriate level of expertise to confirm that it is of an acceptable scientific standard.

# Reviewer report: Reviewer response for version 1

**DOI:** 10.21956/wellcomeopenres.25338.r107360

**Published:** 2024-12-05

**Authors:** Hermina Jakupović

**Affiliations:** 1 University of Copenhagen, Copenhagen, Denmark

Overall comments for the manuscript:

 In summary, this pilot study addresses the quality of health care in the African population – an example of resource-limited environments and a highly heterogenous population, focusing on the feasibility of a coordinated phenotyping and genotyping approach conducted in the Western Cape Province, South Africa, with virtual follow-up. They aim to address the limited knowledge regarding African genetic drivers of disease by analyzing a total of 2,267,346 variants from 459 unrelated participants recruited by using a tiered informed consent model. DNA was collected by buccal swab (and blood samples?), and samples were analyzed using the H3Africa Illumina microarray. Phenotype data, such as data on type 2 diabetes mellitus (T2DM) and severe COVID-19, were obtained from the electronic routine health data of the participants. Data on T2DM and severe COVID-19 were used to demonstrate the feasibility of scale-up and novel health discoveries through nested case-control studies. They reported an extensive ancestry admixture in study participants, with 74.0% of the samples, and 78.6% of the variants passing the quality control. Compared to the control group, 3 and 23 variants (pre- and post-imputation data, respectively) out of the 1,780 published COVID-19 associated variants were significantly associated with COVID-19 cases, whereas for the T2DM, 69 and 419 variants (pre- and post-imputation data, respectively) out of the 2,755 published, were significantly associated with T2DM cases. Indeed, this study addresses an important aspect of gaining much-needed knowledge about previously understudied populations, illustrating what could be possible if the cohort expands in the future, and could potentially be used to inform the local healthcare and public health aspects - provided that genetic analyses are offered to all who can benefit.

Comments for Abstract (and beyond):

1. Please, add the proportion and N of cases and controls, and additionally add the proportion and N of women/men included in the overall study next to the total sample size (N=459), and for cases and controls separately per disease. Provide also information on how many within each sub-strata were a T2DM / COVID-19 case, including any overlaps. This comment is relevant to the whole manuscript, where relevant (mention all this also in the main Methods section of the overall manuscript accompanied by a flow-chart, and again, in the main Results section of the overall manuscript, where briefly describing descriptive characteristics of the study population).
2. Please, provide the relevant information for the follow-up time at the time of the study. As mentioned above, this comment is relevant to the whole manuscript, and there are also possible differences in follow-up times between case and control groups, if possible.
3. Please, provide the relevant information on age distribution as well. As mentioned above, this comment is relevant to the whole manuscript, where relevant, as age is an especially important aspect when diseases such as T2DM and severe COVID-19 are used as endpoints.
4. Abstract Results, please add p-values.
5. To clarify, the sentences could be modified by adding the word “previously”, where mentioning the N of previously published variants for each disease [“Out of *previously* published X associated variants, …”], as some of the readers may not be as familiar with genetics-focused studies, and especially in the abstract part of the manuscript.
6. This is a really minor comment, but I would kindly ask the authors to add commas to the numbers to separate thousands and report % with an equal number of decimals. This comment is relevant to the whole manuscript.

Comments for Introduction:

1. In general, readability and flow are really good, and all chapters are on point.
2. Could the authors kindly also explain to the readers, why T2DM and severe COVID-19 were specifically chosen to demonstrate the feasibility of scale-up and novel health discoveries through nested case-control studies?

Comments for the other parts of the manuscript:

1. The manuscript is properly written and referenced, well explained, and the aim and motivation are clearly stated.
2. I would suggest the authors to change the first chapter in the Results to the one with the population descriptives (whether these observed associations varied among different groups, sex, age, and the obesity status of individuals if available as it is one main risk factor for both disease endpoints, and might have a big effect on observed associations between the case and control groups in this study as well), this is of course just a preference, but I think it also might improve the readability for this section.
3. Methods part on how the final study population was defined would definitely improve by adding the flow-chart to the manuscript (as also mentioned above). In addition to the relevant information on strata (cases/controls, original study sample, how the randomization was performed for the nested case-control study setting?) Please provide full exclusion and inclusion criteria in the self-explanatory figure as well (incl. the health parameters, medication, and all other relevant and included factors here (for phenotypes- and genotype data), with actual numbers of individuals being excluded per each criterion as done in the text.
4. “459 unrelated study participants” – This information (along with other information in the QC sections) could also be part of the flowchart of the Methods, including the N of participants included/excluded during the final selection of the study population.
5. Could the authors please provide the relevant ICD- codes used for T2DM and severe COVID-19, if relevant? What was a positive predictive value for the identification of diabetes cases using the criteria in the study, and what was the criteria? Please clarify. I would kindly ask the authors to touch upon this in the discussion as well, and how this could potentially affect the results. Are also COVID-19 cases from this same data collected from healthcare clients visiting diabetes day clinics at Groote Schuur Hospital? I can see also HIATUS study being mentioned, so I would advise the authors to add these also to the flow-chart, to make it more clear for the readers to understand how the final study population was selected, where the data came from, and which inclusions and exclusions were used, if and how many excluded because they were closely related etc, including the possible overlaps and information on possible randomization.
6. Is the data collected from healthcare clients visiting diabetes day clinics at Groote Schuur Hospital representative to study T2DM in African population, are there socioeconomic differences worth mentioning here for the readers? How about with severe COVID-19?
7. I would advise the authors to focus on the phenotypes of interest and mention the prevalence of T2DM and COVID-19 within the female/male populations and among participants aged 45-64+ years. Are there differences? Here in general, the differences between body weight and T2DM: these might be mediating the effects of the current study. Could the authors elaborate on this? I would also suggest that authors add the effect of age in the discussion, as it would be important to include it, especially as we are focusing on diseases with higher onset in this age group.
8. Gene names: in *italics*.
9. Moreover, just out of interest: an idea for future studies with the data: I would suggest making additional sensitivity analyses separately for women in their post- and premenopausal period of life and to see how the results differ, as the reference groups would also now differ, i.e., possible effects would not be “diluted” here.
10. Table 1: Could the authors provide information on age also separately for male and female case groups and the controls? Do we see any differences there?
11. Table 1: Here again with the DNA sample, also Blood is mentioned but it is not mentioned above in all other sections where only buccal swabs have been. Please, clarify this across the manuscript for consistency.

Is the work clearly and accurately presented and does it cite the current literature?

Yes

If applicable, is the statistical analysis and its interpretation appropriate?

Yes

Are all the source data underlying the results available to ensure full reproducibility?

Yes

Is the study design appropriate and is the work technically sound?

Yes

Are the conclusions drawn adequately supported by the results?

Partly

Are sufficient details of methods and analysis provided to allow replication by others?

Yes

Reviewer Expertise:

Genetic epidemiology, cardiometabolic and endocrine health, obesity, pregnancy and obstetrics, fetal development, brain gliomas, immune development, complex diseases, gene-environment interactions

I confirm that I have read this submission and believe that I have an appropriate level of expertise to confirm that it is of an acceptable scientific standard, however I have significant reservations, as outlined above.

# Response

**Published:** 2024-12-17

**Authors:** Nicki Tiffin

**Affiliations:** South African National Bioinformatics Institute, University of the Western Cape Faculty of Natural Science, Bellville, Western Cape, South Africa

Thank you for your careful review of our manuscript and helpful comments

*Comments for Abstract (and beyond):Please, add the proportion and N of cases and controls, and additionally add the proportion and N of women/men included in the overall study next to the total sample size (N=459), and for cases and controls separately per disease. Provide also information on how many within each sub-strata were a T2DM / COVID-19 case, including any overlaps. This comment is relevant to the whole manuscript, where relevant (mention all this also in the main Methods section of the overall manuscript accompanied by a flow-chart, and again, in the main Results section of the overall manuscript, where briefly describing descriptive characteristics of the study population).* 

**Response: **Thank you for highlighting these points. In the abstract we have now included the proportions of women/men in the overall study. We have provided the numbers of cases and controls for T2DM and severe covid-19 in the abstract. As we wanted to test nested analyses for T2DM and severe COVID-19 as independent outcomes, we did not analyse those with comorbid T2DM and COVID-19, although we recognise the importance if comorbidity and intend to conduct these analyses when the cohort reaches a significant size. We would not like the readers to misinterpret our feasibility study as a complete analysis and the sample size is not yet sufficient to draw conclusions. For this reason, we have not included a flow chart in case it is misconstrued as a summary for a complete study.  This will be drawn up for our first analysis that can demonstrate statistical significance and reliable results.

*Please, provide the relevant information for the follow-up time at the time of the study. As mentioned above, this comment is relevant to the whole manuscript, and there are also possible differences in follow-up times between case and control groups, if possible. Please, provide the relevant information on age distribution as well. As mentioned above, this comment is relevant to the whole manuscript, where relevant, as age is an especially important aspect when diseases such as T2DM and severe COVID-19 are used as endpoints. I would advise the authors to focus on the phenotypes of interest and mention the prevalence of T2DM and COVID-19 within the female/male populations and among participants aged 45-64+ years. Are there differences? Here in general, the differences between body weight and T2DM: these might be mediating the effects of the current study. Could the authors elaborate on this? I would also suggest that authors add the effect of age in the discussion, as it would be important to include it, especially as we are focusing on diseases with higher onset in this age group.*  

**Response: ** We recognise the importance of age distribution and follow-up time for analysis of data from cohorts, and this is particularly important when calculating incidence of specific conditions. Because this is a feasibility study in the early stage of cohort development we have done this analysis for any condition. Once the cohort reaches an appropriate size and we undertake analyses that will yield statistically significant results, we will undertake the necessary univariate and bivariate association analyses to ensure we identify any systematic bias in out nested case-control studies. We envisage conducting multivariate analyses using genomic data in combination with age, sex and multimorbidity data to better understand drivers of disease. It is exactly this kind if analyses for which we believe the final cohort would be best suited, but we believe it would be premature to do these analyses with a limited sample size for this feasibility study. In other studies, we have conducted using health data from the PHDC, we have calculated a median length of time for which individuals have available data of more than 8 years (Osei-Yeboah et al. 2021, PLOS Global Public Health).

*Please, provide the relevant information on age distribution as well. As mentioned above, this comment is relevant to the whole manuscript, where relevant, as age is an especially important aspect when diseases such as T2DM and severe COVID-19 are used as endpoints.* 

**Response: **Thank you for your comment. In the results section and in Table 2, we discuss the age distributions between cases and controls in detail.  

*Abstract Results, please add p-values.*   ​​​​​​​

**Response: **We have added p-values  

*To clarify, the sentences could be modified by adding the word “previously”, where mentioning the N of previously published variants for each disease [“Out of previously published X associated variants, …”], as some of the readers may not be as familiar with genetics-focused studies, and especially in the abstract part of the manuscript.*   ​​​​​​​

**Response: **We have corrected this throughout the manuscript.  

*This is a really minor comment, but I would kindly ask the authors to add commas to the numbers to separate thousands and report % with an equal number of decimals. This comment is relevant to the whole manuscript.*   ​​​​​​​

**Response: **Thank you for the comment, we are working within the editorial policy of the journal which requires metric representation of the numbers.  

*Comments for Introduction: In general, readability and flow are really good, and all chapters are on point.*  

 ​​​​​​​ **Response: **Thank you for the compliment on the manuscript.  

*Could the authors kindly also explain to the readers, why T2DM and severe COVID-19 were specifically chosen to demonstrate the feasibility of scale-up and novel health discoveries through nested case-control studies?*  

 ​​​ **Response: **We selected one non-communicable and one infectious disease that was highly prevalent in our population at the time of the feasibility study. T2DM is a significant public health challenge in with a prevalence of more than 12% in South Africa; and at the time of our feasibility study, our collaborators were prioritising studies on drivers of COVID-19 because it was highly prevalent at the time. We have added this information to the methods section under ‘Identification of cases and controls’.  

*Comments for the other parts of the manuscript:* *The manuscript is properly written and referenced, well explained, and the aim and motivation are clearly stated.* *I would suggest the authors to change the first chapter in the Results to the one with the population descriptives (whether these observed associations varied among different groups, sex, age, and the obesity status of individuals if available as it is one main risk factor for both disease endpoints, and might have a big effect on observed associations between the case and control groups in this study as well), this is of course just a preference, but I think it also might improve the readability for this section.* *Methods part on how the final study population was defined would definitely improve by adding the flow-chart to the manuscript (as also mentioned above). In addition to the relevant information on strata (cases/controls, original study sample, how the randomization was performed for the nested case-control study setting?) Please provide full exclusion and inclusion criteria in the self-explanatory figure as well (incl. the health parameters, medication, and all other relevant and included factors here (for phenotypes- and genotype data), with actual numbers of individuals being excluded per each criterion as done in the text. “459 unrelated study participants” – This information (along with other information in the QC sections) could also be part of the flowchart of the Methods, including the N of participants included/excluded during the final selection of the study population.*   ​​​​​​​

**Response: **As we stated previously this is a feasibility study and we would not like any reader to over-interpret the results. With the feasibility study we are reporting on the successful use of this methodology rather than any specific findings and for this reason we focus on the methodological approach and its implementation. With respect, and after careful consideration of your suggestion, we would prefer to leave the order as it is for this reason. As discussed previously, we have decided not to include a flow chart as we believe this would be more appropriate when we are able to conduct a statistically significant epidemiological analysis which we will do once the cohort reaches an appropriate size. As such, what we present here a methodological paper rather than an epidemiological paper.  

*Could the authors please provide the relevant ICD- codes used for T2DM and severe COVID-19, if relevant? What was a positive predictive value for the identification of diabetes cases using the criteria in the study, and what was the criteria? Please clarify. I would kindly ask the authors to touch upon this in the discussion as well, and how this could potentially affect the results. Are also COVID-19 cases from this same data collected from healthcare clients visiting diabetes day clinics at Groote Schuur Hospital? I can see also HIATUS study being mentioned, so I would advise the authors to add these also to the flow-chart, to make it more clear for the readers to understand how the final study population was selected, where the data came from, and which inclusions and exclusions were used, if and how many excluded because they were closely related etc, including the possible overlaps and information on possible randomization.*  

 ​​​​​​​ **Response: **With the use of the routine health data from the PHDC, rather than using ICD-10/11 codes, the ascertainment of both T2DM and COVID-19 the PHDC uses a series of algorithms to ascertain these conditions in individuals. This is described in the Methods section (‘Identification of cases and controls'). In both these conditions, the laboratory data provide a strong basis for diagnosis (HbA1c and PCR tests). In addition, the HIATUS study had a clinical diagnosis of severe COVID-19 provided by a healthcare professional and described for that study in the referenced publication. This clinical observation is of course additional to the PHDC data, but in our collaborative model we anticipate in the future collaborating with other studies where additional data may be leveraged, so we believe this supports our collaborative approach to grow the cohort, rather than detracting from it. Of course for some conditions the process of ascertainment might be more difficult using PHDC data, and the PHDC at the Western Cape Department of Health and Wellness works to validate the ascertainment algorithms as they are developed. As such, a sensitivity/specificity analysis and calculation of a PPV of the PHDC algorithms are beyond the scope of this feasibility study where we are testing the methodology to successfully build a cohort linking genomic and routine health data;and we do not have sufficient data to conduct an analysis of the effectiveness of the PHDC ascertainment algorithms additional to those undertaken by the PHDC analysts.  

*Is the data collected from healthcare clients visiting diabetes day clinics at Groote Schuur Hospital representative to study T2DM in African population, are there socioeconomic differences worth mentioning here for the readers? How about with severe COVID-19?*   ​​​​​​​ **Response: **In this feasibility study we focused on genomic drivers of disease and we believe genetic variation is not affected by socio-economic status. In addition, the PHDC does not collect socio-economic data so these are not available to us. In future collaborative studies, however, this cohort will be well positioned to incorporate socio-economic data collected in other studies and from other data sources in combination with health data and genomic data in multivariate analyses.  

*​​​​​​​Gene names: in italics.* ​​​​​​​

**Response: **Thank you for flagging this, it has been done.

*Moreover, just out of interest: an idea for future studies with the data: I would suggest making additional sensitivity analyses separately for women in their post- and premenopausal period of life and to see how the results differ, as the reference groups would also now differ, i.e., possible effects would not be “diluted” here.*  

 ​​​​​​​ **Response: **Thank you for this interesting suggestion, we will definitely seek collaborators working in this area for future studies when our cohort is large enough.  

*Table 1: Could the authors provide information on age also separately for male and female case groups and the controls? Do we see any differences there?*  ​​​​​​​

**Response: ** We appreciate that this stratified analysis would be of interest but we will only be able to undertake this in a meaningful way when we have a larger cohort.  

*Table 1: Here again with the DNA sample, also Blood is mentioned but it is not mentioned above in all other sections where only buccal swabs have been. Please, clarify this across the manuscript for consistency.*   ​​​​​​​

**Response: **For this feasibility study we used DNA from blood samples collected as part of the HAITUS study as well as de novo collection and preparation of DNA from buccal swabs. This provided us with an opportunity to test the feasibility of using DNA samples prepared from different studies to ensure that the results are comparable. Whilst this adds to the complexity of the methodology we believe it was important to test the efficacy and success of this approach as it will allow us to grow the cohort through both de novo recruitment and future collaborations. We believe that this collaborative approach will ensure that the cohort we are building will become a collaborative and uselful resource for health researchers across the Western Cape Province of South Africa. It was thus important for us to demonstrate the flexibility inherent in the design of the cohort. In the manuscript we have aimed to clarify the different methodologies used and compare methodological results for DNA from blood samples and buccal swabs.

# Reviewer report: Reviewer response for version 1

**DOI:** 10.21956/wellcomeopenres.25338.r107363

**Published:** 2024-11-25

**Authors:** Emilio Di Maria

**Affiliations:** 1 University of Genoa, Genoa, Italy

The article reports a pilot study to demonstrate the feasibility of a coordinated phenotyping and genotyping approach conducted in the Western Cape Province, South Africa.

 The study addresses a very important issue, that is the quality of health care in the African context. Closing the gap between the affluent and the less resourced area is a priority for global health. The implementation of genomics may drive improvements of care processes towards personalised medicine, provided that genetic analyses are offered to all who can benefit.

 Moreover, there is an emerging need for a representation of diverse population in clinical research, where the individuals of African ancestry are under-represented.

 The manuscript is properly written and referenced.

 The ethical aspects are particularly relevant in the present study and were adequately addressed [despite the ethics and consent chapter is located at the end of the article according to the recommended structure].

 I have few substantial remarks, namely on the interpretation of the study findings.

 Methods:

 The first paragraph reports that participants were enrolled at diabetes day clinics. Several paragraphs below, it turns out that participants from the study on HIV and TB in COVID-19 were also enrolled. For an effective description of the study cohort, settings and procedure could be grouped in the same section.

 As well, the procedures for DNA extraction from both buccal swab and blood should be in the same section. Furthermore, as standard methods were applied, the detailed description of the laboratory procedures could be shortened.

 Conversely, the readers who are not familiar with genotyping techniques would be happy to find a brief description of the H3Africa array, though it is a commercial kit.

 Discussion:

 - The authors conclude that the pilot study demonstrated that the approach was cost-effective. In fact, the transferability of the pilot protocol into mainstream medicine requires a structured assessment of several domains, including economics. However, the article provided no measure of cost-effectiveness – by the way, neither estimation of costs was given. Affordability may be claimed, but cost-effectiveness looks an overstatement.

 - Given the settings of recruitment, participants are supposed to be biased towards diabetes and infectious diseases. This is acceptable for a pilot study but should be acknowledged as a limitation. Similarly, it is questionable that the ‘cohort may be used as disease-agnostic resource’ [page 13].

 Minor remarks:

 All gene names should be in italics – in tables too.

 A few typos should be corrected – e.g. page 11 5 ^th^ par, page 12 2 ^nd^ par.

Is the work clearly and accurately presented and does it cite the current literature?

Yes

If applicable, is the statistical analysis and its interpretation appropriate?

Yes

Are all the source data underlying the results available to ensure full reproducibility?

Yes

Is the study design appropriate and is the work technically sound?

Yes

Are the conclusions drawn adequately supported by the results?

Partly

Are sufficient details of methods and analysis provided to allow replication by others?

Yes

Reviewer Expertise:

Clinical Genetics, Genetic Epidemiology, Health Technology Assessment, Health Equity.

I confirm that I have read this submission and believe that I have an appropriate level of expertise to confirm that it is of an acceptable scientific standard, however I have significant reservations, as outlined above.

# Response

**Published:** 2024-12-17

**Authors:** Nicki Tiffin

**Affiliations:** South African National Bioinformatics Institute, University of the Western Cape Faculty of Natural Science, Bellville, Western Cape, South Africa

*The article reports a pilot study to demonstrate the feasibility of a coordinated phenotyping and genotyping approach conducted in the Western Cape Province, South Africa. The study addresses a very important issue, that is the quality of health care in the African context. Closing the gap between the affluent and the less resourced area is a priority for global health. The implementation of genomics may drive improvements of care processes towards personalised medicine, provided that genetic analyses are offered to all who can benefit. Moreover, there is an emerging need for a representation of diverse population in clinical research, where the individuals of African ancestry are under-represented. The manuscript is properly written and referenced.* *The ethical aspects are particularly relevant in the present study and were adequately addressed [despite the ethics and consent chapter is located at the end of the article according to the recommended structure].*  

**Response: **Thank you for your review and helpful comments about this manuscript  

*Methods:*

*The first paragraph reports that participants were enrolled at diabetes day clinics. Several paragraphs below, it turns out that participants from the study on HIV and TB in COVID-19 were also enrolled. For an effective description of the study cohort, settings and procedure could be grouped in the same section. As well, the procedures for DNA extraction from both buccal swab and blood should be in the same section.*  

**Response: **Thank you for highlighting that the flow for this section could be improved, we have rearranged the methods section accordingly, grouping the settings and procedure sections as requested.  

*Furthermore, as standard methods were applied, the detailed description of the laboratory procedures could be shortened.*  

**Response: **Thank you for your observation, we were specifically requested by the Journal to provide this high level of detail in order to meet their requirements.  

*Conversely, the readers who are not familiar with genotyping techniques would be happy to find a brief description of the H3Africa array, though it is a commercial kit.*  

**Response: ** Thank you for highlighting this, we have added some more detail in the section on genotyping.

*Discussion:The authors conclude that the pilot study demonstrated that the approach was cost-effective. In fact, the transferability of the pilot protocol into mainstream medicine requires a structured assessment of several domains, including economics. However, the article provided no measure of cost-effectiveness – by the way, neither estimation of costs was given. Affordability may be claimed, but cost-effectiveness looks an overstatement.* **Response: **Thank you for highlighting that the term cost-effective has specific economic meaning. We have replaced it with the phrase “affordable in a resource limited context”.  

*Given the settings of recruitment, participants are supposed to be biased towards diabetes and infectious diseases. This is acceptable for a pilot study but should be acknowledged as a limitation. Similarly, it is questionable that the ‘cohort may be used as disease-agnostic resource’ [page 13].*  

**Response: **Thank you for highlighting this. We recognise that as a feasibility study we have not yet established a complete, large, disease agnostic cohort. What we seek to demonstrate here is that this approach can be used successfully to generate such a cohort. We have deliberately recruited with a bias towards common diseases in our population to ensure early in the establishment of our cohort that we can successfully conduct disease specific analyses, as we demonstrate here. As the cohort grows in size, recruitment will indeed be from the general population, and we are already recruiting participants from dentistry clinics to expand beyond the initial disease focus. We have added a paragraph in the discussion that clearly articulates this.  

*All gene names should be in italics – in tables too.*  

**Response: **Thank you for this note, we have implemented this throughout the manuscript.  

*A few typos should be corrected – e.g. page 11 5 ^th^ par, page 12 2 ^nd^ par.*  

**Response:**Thank you for picking these up, we have corrected them. Thank you for flagging that the reference is in lowercase in the downloaded and online version of the manuscript. We will flag this with the editors.
